# The Nexus Between Diabetes and Depression: A Narrative Review

**DOI:** 10.7759/cureus.25611

**Published:** 2022-06-02

**Authors:** Salma Habib, Sai Lahari Sangaraju, Daniela Yepez, Xavier A Grandes, Ramya Talanki Manjunatha

**Affiliations:** 1 Internal Medicine, Institute of Applied Health Sciences (IAHS), Chittagong, BGD; 2 Research, People’s Education Society (PES) Institute of Medical Sciences and Research, Kuppam, IND; 3 General Medicine, Universidad Catolica de Santiago de Guayaquil, Guayaquil, ECU; 4 Graduate Medical Education, Kempegowda Institute of Medical Sciences, Bangalore, IND

**Keywords:** diabetes-related distress, impact of diabetes and depression on life, diabetes and depression prevalence, depression screening, comorbid diabetes and depression management, type 2 diabetes and depression, type 1 diabetes and depression, depression, diabetes and depression

## Abstract

Comorbid diabetes and depression are a significant public health burden as the consequences of both diseases are worsened by each other. In this study, we have compiled and analyzed findings from various studies to demonstrate that diabetes has a strong association with depression. Both have a significant impact on the quality of life, although the exact mechanisms through which these two chronic diseases affect each other remain unknown. This article discussed the shared etiological factors of comorbidity between diabetes and depression, including physiological (e.g., deregulation of the hypothalamic-pituitary-adrenal (HPA) axis, sympathetic nervous system (SNS) overactivity, microvascular dysfunction, arterial stiffening, inflammation, and cytokines), behavioral (e.g., diet and lifestyle modifications), and environmental (e.g., childhood adversity, poverty, and neighborhood environment). Included data from a range of settings have suggested that the prognosis of both diabetes and depression, in terms of complications, treatment efficacy, morbidity, and mortality, is worse for either disease when they occur concurrently than individually. The implication for the physical, mental, and social well-being of depression in diabetes causes poor self-care and adherence to medical treatment. This article also highlights the importance of regular screening and prompts the treatment of comorbid diabetes and depression with pharmacotherapy, face-to-face psychotherapy, and non-face-to-face models of alternative psychological interventions, including information and communication technologies (ICTs), computer-based diabetes self-management interventions, and digital mental health intervention, to improve the outcomes of both diseases.

## Introduction and background

Diabetes is one of the widely spreading chronic diseases globally [1]. According to the International Diabetes Federation, approximately 536.6 million people are affected by diabetes worldwide, and the number is predicted to increase to 783.2 million by 2045 [1]. In 2021, the Centers for Disease Control and Prevention (CDC) in the United States (US) estimated that roughly 37 million (11.3%) of the population were affected by diabetes [2]. Diabetes is characterized by chronically elevated blood glucose levels due to inadequate insulin production by autoimmune-mediated destruction of the pancreatic beta-cells (β-cells) and insufficient use of insulin by the body [3]. Depending on pathophysiology, diabetes is classified into four categories, as presented in Table 1 [3].

**Table 1:** Classification of Diabetes Mellitus

Types of Diabetes Mellitus	Pathophysiology
Type 1 diabetes mellitus (T1DM)	Autoimmune-mediated β-cell destruction
Type 2 diabetes mellitus (T2DM)	Insufficient insulin production or insulin resistance
Gestational diabetes mellitus (GDM)	Pregnancy-induced glucose intolerance
Others	Drugs, chemicals, and genetic and pancreatic disorders

Compared to White and Native American ethnic groups, the prevalence of diabetes is higher in African-American women [4]. By the year 2050, diabetes prevalence is expected to triple in African-Americans and double in Whites [5]. Along with short-term (e.g., insulin-related hypoglycemia) and long-term (e.g., cardiovascular disease, neuropathy, nephropathy, and retinopathy) complications, diabetes has detrimental effects on mental health, chiefly among them is depression [6,7]. Patients with depression exhibit persistent depressed mood for two weeks along with at least five of the following symptoms: decreased interest or pleasure, altered sleep pattern with insomnia or hypersomnia, changes in appetite with weight loss or gain, feelings of guilt or worthlessness, lack of energy, poor concentration, suicidal thoughts, and psychomotor agitation and retardation [7]. The depression rate in developed and developing countries is 15% and 11%, respectively [8]. In a meta-analysis, Chen et al. showed that the prevalence of depression was positively correlated with prediabetes (odds ratio (OR): 1.11), undiagnosed diabetes (OR: 1.27), and previously diagnosed diabetes (OR: 1.80), compared with normoglycemic individuals [9]. Diabetes and depression have common symptoms, including change in appetite, low energy, weight change, and poor concentration [10]. However, depression frequently persists as unidentified and unexplored [11]. One study in Europe demonstrated that depression is not typically discussed during routine diabetes-related visits, presumably due to patients and physicians emphasizing somatic symptoms and complications more than psychological symptoms associated with diabetes [12]. Consequently, clinical outcomes are declining due to inadequate management of depression, leading to nonadherence to lifestyle modifications, medication noncompliance, and poor glycemic control; this ultimately increases mortality [7]. Depression and anxiety are the fourth and diabetes is the eighth reason for disability-adjusted life years (DALYs) in developed countries [13]. Psychosocial factors, including depression, stress, and poor social support, may lead to or worsen diabetes [14]. This review article aims to understand the association between diabetes and depression and highlight the importance of diagnosing and managing depression in diabetes to improve quality of life.

## Review

Pathophysiological association between diabetes and depression

Preclinical data show that metabolic dysfunction may be caused by behavioral, genetic, and physiological factors in neuroimmunological and neuroendocrinal alterations and microvascular dysfunctions, which can be long-lasting and play a significant role in comorbid diabetes and depression [15,16].

Physiological Factors

Depression and T2DM share common biological origins through hypothalamic-pituitary-adrenal (HPA) axis deregulation, autonomic nervous system (ANS) hyperactivity, and inflammatory processes [17]. In response to physiological or psychological stressors, the HPA axis is activated, resulting in the secretion of corticotrophin-releasing hormone (CRH) from the hypothalamus, which stimulates the anterior pituitary gland to release adrenocorticotropic hormone (ACTH). ACTH then stimulates the release of cortisol from the adrenal gland [18]. Similarly, chronic stress over-activates the sympathetic nervous system (SNS) and leads to an increase in catecholamine release [19]. High cortisol and catecholamine levels impair insulin binding to its receptor, leading to insulin resistance and the development of hyperglycemia [20]. For instance, Weber et al. found that insulin sensitivity was impaired in 26 patients with comorbid depression and hypercortisolism [20]. Additionally, Vogelzangs et al. conducted a study on 867 patients aged 65 and above and demonstrated a significant interaction (p=0.003) between depression and hypercortisolism, increasing the risk for metabolic syndrome [21].

Hyperglycemia is a possible reason for hippocampal atrophy. There is an inverse relationship between blood sugar level and hippocampal volume, which is detected by glycated hemoglobin (HbA1c) level [22]. Prolonged hyperglycemia or fluctuating glucose causes neuronal damage by activating the polyol pathway, which induces oxidative stress and increases the formation of advanced glycation end products (AGEs) [23]. Using the Mini-International Neuropsychiatric Interview and the nine-item Patient Health Questionnaires (PHQ-9), van Dooren et al. conducted a cross-sectional study and found that AGEs were associated with somatic and cognitive symptoms of depression [24]. Depression is also associated with neurodegenerative processes, especially in the prefrontal cortex and hippocampus [25]. Several studies suggest that chronic stress induces immune dysfunction and increases the production of inflammatory cytokines directly or through the HPA axis or SNS [26]. High amounts of inflammatory cytokines impair insulin sensitivity by binding with pancreatic β-cells and promote the development of T2DM [26]. Additionally, inflammatory cytokines may alter many pathophysiological domains that characterize depression, including neurotransmitter signaling and neuroendocrine and neurosynaptic transmission [27]. Furthermore, vascular damage via microvascular dysfunction and arterial stiffening mediate a linkage between diabetes and depression [28]. Microvascular dysfunction impairs glucose and insulin intake at a cellular level and may cause insulin resistance [29]. Recently, van Agtmaal et al. performed a meta-analysis on a 40-year or older population and demonstrated increased odds of developing late-life depression in patients with both peripheral and cerebral microvascular dysfunction (OR: 1.19; 95% confidence interval (CI): 1.09-1.30) [30].

Similarly, T1DM has a biological link with depression, which is evidenced by the study of Korczak et al. [31]. Suffering from a long-lasting disorder from a very early age while personality is also developing is a reason for psychological stress and might increase the susceptibility to depression in T1DM [31].

**Figure 1:**
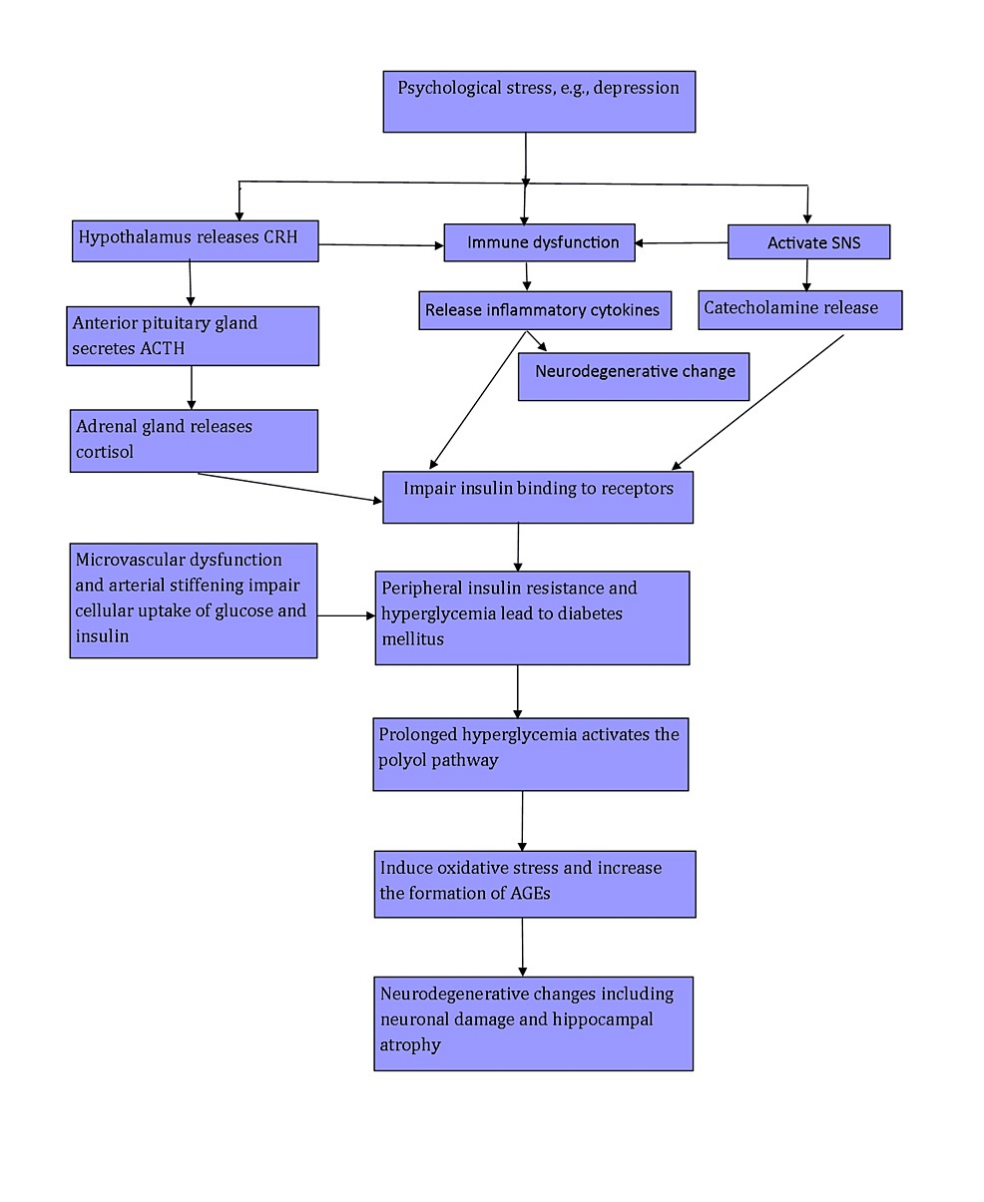
Physiological Association Between Type 2 Diabetes Mellitus (T2DM) and Depression CRH: corticotrophin-releasing hormone, ACTH: adrenocorticotropic hormone, SNS: sympathetic nervous system, AGEs: advanced glycation end products Image credits: Salma Habib

A summary of the physiological association between T2DM and depression is listed in Figure 1.

Behavioral Factors

Depression may be a consequence of behavioral factors such as improper dietary habits, inadequate physical activity, and altered sleep patterns [16]. These altered behavioral factors govern glucose metabolism and independently influence nutrition and lifestyles, predisposing individuals to T2DM [32].

Diet: Compared to nondepressed individuals, depressed patients are more prone to poor dietary habits such as refined sugar and saturated fat than a healthy diet, including fruits and vegetables, further worsening their diabetes control [33]. Currently, the US government highly emphasizes the quality of the food; as a result, instead of recommending limitations on the amount of carbohydrate, protein, and fat in foods, they are centered on the types or patterns of food, including a healthy US-type eating pattern, a vegan or Mediterranean diet [34].

Obesity: There is a significant relationship between obesity, diabetes, and depression, as depression risk is 55% higher in obese people and obesity risk is 58% higher in depressed individuals [35]. In the meta-analysis of Luppino et al., a bidirectional association between obesity and depression was found, more pronounced among Americans than among Europeans (p=0.05). The study showed that obesity increased the risk of the onset of depression (unadjusted OR: 1.55; 95% CI: 1.22-1.98; p<0.001). Similarly, depression increased the risk of developing obesity (OR: 1.58; 95% CI: 1.33-1.87; p<0.001) [36].

Physical activity: All types of physical activity are inversely related to diabetes risk. An epidemiological study showed that diabetes relative risk was reduced even with a low level of physical activity, such as 5-7 hours of leisure time and vigorous or low-intensity exercise per week [37].

Socioeconomic status: Low socioeconomic status has 40%-60% more risk of developing diabetes than high socioeconomic group [38]. Social instability, e.g., lower social cohesion and social capital, increased violence, decreased residential stability, and reduced walkability, plays a significant role in comorbid diabetes and depression [39].

Sleep: Altered sleep and circadian rhythm may lead to depression and T2DM [40]. In a recent meta-analysis of prospective studies, Shan et al. demonstrated that diabetes risk was minimal with 7-8 hours of sleep per day, and for each one-hour sleep deprivation, the risk was increased by 9% [41]. Similarly, Rao et al. conducted a randomized crossover trial in sleep laboratories that showed a decrease in insulin sensitivity by 29% after sleep restriction for five days [42].

Environmental Factors

Environmental factors, ranging from intrauterine life to neighborhood surroundings, may increase the risk of comorbid diabetes and depression [43]. Evidence suggests that an intrauterine environment and low birth weight (LBW) put an individual at risk of developing diabetes in childhood, adolescence, and adulthood. High cortisol level secondary to maternal stress causes fetal overexposure to cortisol, which may predispose the individual to stress-related and metabolic disorders [44,45]. Nevertheless, there is an inconclusive relationship between a poor intrauterine environment and risk for adult depression [46,47]. Thompson et al. included 882 full-term birth newborns in their study and measured weight at birth at one year. Depression screening at 68 years of age showed a significantly increased prevalence of depression in LBW individuals [46]. In contrast, Colman et al. conducted a prospective cohort study in Canada on 3,732 patients aged 12-15 years and showed an inconsistent relationship between LBW and depression; however, the study mentioned that people who lead a stressful life might have a higher chance of developing depression later in life [47].

Other environmental factors, including childhood adversity, poverty, neighborhood environment, traffic, and noise, increase the susceptibility to comorbid diabetes and depression [39]. A good neighborhood and family support with encouragement and praise increase awareness in the children and adolescents to involve in more physical activity that could change them in a healthy mood and eventually improve their quality of life [48,49].

Genetic Factors

Several studies demonstrated that diabetes has little or no association with depression at the genetic level [50,51]. A study on middle-aged male twins in Vietnam on a sample population of 1,237 by Scherrer et al. found no correlation between diabetes and depression (r=0.19; 95% CI: 0.00-0.46) on the genetic level [50]. In comparison, Samaan et al. performed a cohort study on 17,404 patients from multiethnic backgrounds with a risk of T2DM, including 3,209 depression cases and 14,195 without depression. A total of 20 single-nucleotide polymorphisms (SNPs) associated with T2DM were genotyped using the cardiovascular gene-centric 50-K SNP array. Of the 20 SNPs, 12 were associated with T2DM (p=0.048). However, the 20 SNPs were not associated with depression (p=0.09). The study concluded that genetically T2DM and depression had no association [51].

**Figure 2:**
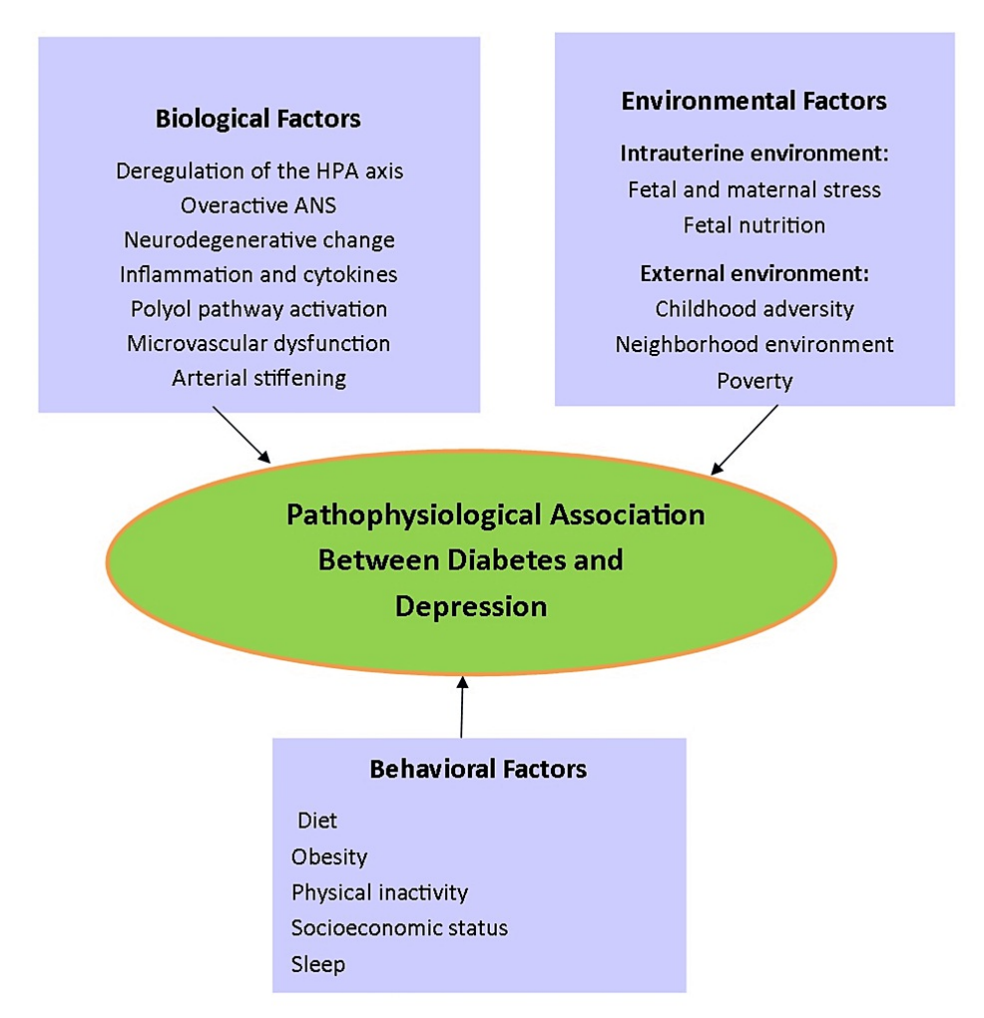
Pathophysiological Factors in Diabetes and Depression HPA axis: hypothalamic-pituitary-adrenal axis; ANS: autonomic nervous system Image credits: Salma Habib

An overview of the pathophysiological factors is shown in Figure 2.

Drug effects on comorbid diabetes and depression

Apart from previous factors discussed earlier, antidepressants (ADMs) also increase diabetes risk in depression; whether this relationship is causative remains unproven [52]. A recent study was observed by Pan et al. on adult diabetic males from the Health Professionals Follow-up Study and diabetic females from the Nurses’ Health Study I and II over three years that put forwarded into consideration that using selective serotonin reuptake inhibitors (SSRIs) or tricyclic antidepressants (TCAs) increased the incidence risk of T2DM [53]. Nevertheless, the individual properties of ADMs may have a different effect on weight and glycemic level maintenance as the study by McIntyre et al. in 2005 indicated that long-acting SSRIs fluoxetine and nonselective hydrazine monoamine oxidase inhibitors (e.g., phenelzine) improved glycemic control and insulin sensitivity; however, noradrenergic ADMs (e.g., desipramine) caused hyperglycemia, and dual-mechanism ADMs (e.g., duloxetine and venlafaxine) did not affect glucose homeostasis [54]. Recently, second-generation or atypical antipsychotics (aripiprazole, olanzapine, and quetiapine) are new to depression treatment [55]. As an augmentation (or the addition of a second agent to ADMs), these atypical antipsychotics show an outstanding improvement [55]. As a result, these atypical antipsychotics are applied widely in depression, and the rate increased from 4.6% to 12.5% in 10 years [56]. However, while treating a patient with diabetes or a higher risk of diabetes, the consequences of the atypical antipsychotic should be kept in mind as these medications escalate the risk of obesity and diabetes by increasing appetite and altering metabolic conditions through high fasting triglyceride levels and insulin resistance [57].

Simultaneously, antidiabetic treatment might cause depression. A cross-sectional study by Berge et al. on 21,845 participants concluded that patients with orally treated diabetes in their 40s had an almost double association with depression compared with patients in their 70s (OR: 1.96; 95% CI: 1.35-2.83) [58].

Prevalence of comorbid diabetes and depression

The prevalence rate of depression is almost three times higher in T1DM and two times higher in T2DM [59]. The study by Anderson et al. suggested that people with diabetes suffered from clinically relevant depressive symptoms at least twice (OR: 2, 95% CI: 1.8-2.2). They performed a meta-analysis from 42 studies on controlled nondiabetic versus uncontrolled groups (21% versus 30%), in the clinical versus community settings (32% versus 20%), on diabetic females versus males (28% versus 18%), and self-reported depression versus structured interview (31% versus 11%) [60]. Whether the prevalence rate depends on the types of diabetes remains unclear. Ali et al. meta-analyzed 10 cross-sectional studies on 51,331 populations and concluded that T2DM had a significantly high prevalence of depression (17.6% versus 9.8%; OR: 1.6; 95% CI: 1.2-2), and it was higher in diabetic females (23.8%) than in diabetic males (12.8%) [61]. For T1DM, Gendelman et al. studied 458 people with diabetes and 546 without diabetes for three years and indicated a higher prevalence rate of depression in T1DM than in individuals without T1DM for males and females (males: 25.5% versus 11.6%; females: 37.9% versus 20.5%) [62]. However, several studies showed a lower depression prevalence rate in diabetic patients, such as a cross-sectional population-based study performed by Holt et al. on 2,997 patients with T2DM. They reported a lower prevalence rate in both T2DM and non-T2DM, which was 3.8% and 5%, respectively, but they found a significant association between them [63]. In comparison, in the systematic review by Barnard et al., it was unclear whether the depression prevalence rate was high in T1DM. In controlled studies, the prevalence rate of depression in diabetic patients versus nondiabetic patients was 12% versus 3.2%, but in studies with no control group, clinical depression was 13.4% [64]. Furthermore, a few studies have reported a similar risk of developing depression in T1DM and T2DM. Recently, a retrospective cohort study on 31,635 people with T2DM and 57,141 without T2DM conducted by Brown et al. reported an almost similar incidence of depression in patients with and without T2DM (6.5% versus 6.6%) [65]. Similarly, Pouwer et al. conducted a study in the Netherlands and mentioned that the prevalence rate of depression was almost similar in patients with T1DM (33%) and T2DM (37%) [66].

Studies on synergistic diabetes and depression are contradictory to individuals suffering from only depression as depressive symptoms remain only for 8-12 weeks in the general population; however, in comorbid diabetes and depression, depressive symptoms are more persistent and recurrent [43]. For instance, Mueller et al. have undertaken a longitudinal prospective study on 485 sample populations over 15 years and found that almost 58%-85% of people experienced relapsing and recurrence depressive symptoms [67].

Impact of comorbid diabetes and depression

The impact of comorbid diabetes and depression appears to be additive, and both combined have higher negative consequences in healthcare than diabetes and depression alone [68]. Suffering from long-standing diseases such as diabetes may develop distress that turns out into emotional distress [69]. This emotional distress often does not approach a certain level to diagnose it as a psychiatric disease. However, a norm-glycemic state can be maintained by improving emotional distress [70]. Diabetes-related emotional distress often causes nonadherence to treatment and lack of self-care; both have a negative impact on diabetic care [71]. For example, a meta-analysis of 47 independent samples, including over 17,000 adults with T1DM and T2DM, performed by Gonzalez et al., found that depression was associated with a lack of treatment adherence and self-management of diabetes [71]. Besides, depressed individuals have an almost double chance of missing antidiabetic drugs; as a result, they might be considered nonreliable for their diabetic management [35]. Gonzalez et al. studied 70 sample populations with an HbA1c of 8.3% (SD: 1.7) and assessed medication use from self-reported HbA1c levels and a medication event monitoring system (a bottle cap that records medication adherence). The results showed that subjects with severe depression and T2DM overstated the number of missed medications [72]. A diabetic individual has to follow a strict regimen of insulin injections, a regular balanced diet, and exercise, which are challenging for the pediatric age group [73]. Parents provide support for the continuation of diabetic care. They might face psychological distress such as stress and anxiety or depression, which further impair the glycemic control of their diabetic children [74]. Whittemore et al. performed a systematic review in 2012 over four years and reported that parental distress at the time of T1DM diagnosis was 33.5%, and four years later, it reached 74% [75].

Effect of depression on morbidity and mortality in diabetes

Depression increases complications or long-term effects in diabetes by altering glycemic control and developing insulin resistance and a more severe diabetic course, including an increased risk of microvascular and macrovascular complications [76]. Chronic hyperglycemia leads to stroke, coronary artery disease, cerebrovascular disease, diabetic retinopathy, nephropathy, and neuropathy [6]. A prospective cohort study carried out by Lin et al. on 4,623 primary care patients over five years rested upon the conclusion that depression causes significantly higher risks of adverse microvascular (hazard ratio (HR): 1.36; 95% CI: 1.05-1.75) and macrovascular (HR: 1.24; 95% CI: 1-1.54) outcomes [77]. Diabetes is a chronic disease; when concurrent with other chronic illnesses (i.e., arthritis, cardiovascular disease, lung disease, stroke, and cancer), the depression rate becomes 2.5 times higher than in non-chronic illness individuals, especially in older adults [70]. As per the meta-analysis of van Steenbergen-Weijenburg et al. of 596 (63%) patients with T2DM in the outpatient clinic, patients with T2DM with two or more complications (OR: 2.23; 95% CI: 1.02-2.94), including neuropathy (OR: 1.7; 95% CI: 1.10-2.77) and nephropathy (OR: 1.68; 95% CI: 1.00-2.48), had more than twofold increase in the risk of depression [78]. Persons with depression in diabetes have poorer cognition and higher dementia risk than those with only diabetes, as evidenced by the study of Chow et al. [79]. They performed an observational meta-analysis on 10 studies from August 2015 to June 2021 (six years) on the adult population and found that persons with comorbid diabetes and depression had worse cognition (SMD: -0.77 (-1.33, -0.20)) and greater dementia risk (HR: 1.82 (1.79, 1.85)) than persons with only diabetes [79]. Although these data cannot speak to causality, the consistency of findings linking depressive symptoms to poor diabetes health outcomes suggests that elevations should be seen as a marker of increased health risk; more longitudinal studies are needed to understand this causal relationship [35].

Cardiovascular causes have a significant role in the mortality of comorbid diabetes and depression [80]. For instance, Egede et al. completed a study over eight years in a sample population of 10,025 participating in the National Health and Nutrition Examination Survey (NHANES) to detect the effect of depression on all-cause mortality secondary to coronary heart disease (CHD) and found a higher mortality rate in individuals with comorbid diabetes and depression than in individuals with diabetes or depression alone [80]. Due to more sick calls, hospital admission, or extended hospital stays, health expenditure increases 4.5-fold in managing both diseases [81,82]. In 2021, the global diabetes-related health expenditures were estimated at 966 billion USD and were projected to reach 1,054 billion USD by 2045 [1].

Management of comorbid diabetes and depression

Diagnostic Approach With Screening

Concurrent diagnosis and treatment of diabetes and depression should be needed to reduce complications and overall health burden [16]. In a study, Li et al. reported that depression remains underdiagnosed and untreated in 45% of diabetic patients [11]. A simple screening of depression in a regular diabetic follow-up might improve the outcomes of both diseases [16]. The most commonly used depression screening questionnaires for people with diabetes are the PHQ-9, Beck Depression Inventory (BDI), Center for Epidemiologic Studies Depression Scale (CES-D), and Hospital Anxiety and Depression Scale (HADS) [83]. The PHQ-9 is the most susceptible and validated screening test for depression evidenced by the study of Kroenke et al., which examined over 9,740 patients from three primary care and one obstetrics-gynecology clinic and found that the PHQ-9 was a highly validated screening test for depression in the clinical settings [84]. Nonetheless, some overlapping somatic symptoms, including sleep patterns, altered appetite, and fatigue in diabetes and depression, often complicate the early diagnosis of depressive symptoms or misdiagnosed depression [10]. The questionnaires that rely heavily on these symptoms may overestimate the probability of depression [85]. A psychiatric evaluation should be needed to make a positive depression screening more reliable [16].

Therapeutic Approach

In diabetic patients, depression can be treated by several pharmacological and psychological treatments, such as cognitive-behavioral therapy (CBT) [86]. The same importance is required for treating both diseases because inadequate depression treatment in diabetic patients is reflected as insufficient blood glucose monitoring or poor diabetes control with significant complications [43].

Pharmacotherapy

Evidence has suggested that ADMs generally have consistently significant effects on depression amelioration in adults with diabetes [87]. Baumeister et al. conducted two studies; one study used ADMs in five trials on 238 diabetic patients and showed that ADMs had effective glycemic control as HbA1c of -0.4% (95% CI: -0.6 to -0.1; p=0.002), and in another study, eight trials on 377 depressed participants for 3-6 months found a moderate beneficial effect of ADMs on short-term depression [87]. However, several studies implied substantial gaps in the evidence for the effectiveness of treating depressive symptoms with SSRIs in glycemic control. The study by Lustman et al. evaluated 26 T1DM and 34 T2DM patients with daily doses of fluoxetine (up to 40 mg/day) for an eight-week randomized placebo-controlled double-blind trial and found that depressive symptoms were significantly reduced in patients treated with fluoxetine compared with those receiving placebo (BDI: -14.0 versus -8.8, p=0.03; Hamilton Depression Rating Scale (HAMD): -10.7 versus -5.2, p=0.01) [88]. In contrast, the study of Paile-Hyvärinen et al. indicated that paroxetine had modest and short-duration glycemic benefits without significantly impacting depression. The study was conducted on 49 mildly depressed primary care outpatients aged 50-70 years for six months with paroxetine 20 mg [89]. Besides, the study of Katon et al. demonstrated that in diabetic patients treated with combined pharmacological and psychological treatment, depression was alleviated without significantly impacting glycemic control [90].

As per efficacy, almost all ADMs have a similar effect on depression. Therefore, ADM selection highly depends on patient preference, effectiveness, and the side effects of ADMs on individuals [91]. A meta-analysis by Serretti et al. aggregating data from 116 studies found a higher risk of weight gain and obesity-related illnesses such as diabetes mellitus, hypertension, and coronary heart disease in ADMs, including mirtazapine paroxetine and amitriptyline. In contrast, some weight loss was noticed with fluoxetine and bupropion [92].

Psychotherapy

CBT and rational therapies help depressed individuals improve negative thinking about themselves, their future, and everyday experiences [35]. In a randomized controlled trial (RCT), Lustman et al. assigned 51 depressed T2DM patients for 10 weeks. Immediately after the completion of the acute phase of treatment and again at six months, the proportion of remitted patients (BDI ≤ 9) was significantly higher in the CBT-receiving group than in the control treatment group (85% versus 27.3% (p<0.001) and 70% versus 33.3% (p=0.03), respectively) [93]. In comparison, the RCT of Snoek et al. on 86 T1DM patients with HbA1c ≥ 8 followed up for one year showed that CBT significantly declined HbA1c level in T1DM patients (p=0.03) [94]. Besides, evidence has suggested that CBT has a better role than ADM in depressed patients regarding glycemic control [95]. In an RCT in Germany, Petrak et al. included 251 T1DM and T2DM patients from 70 secondary care centers with HbA1c > 7.5% and added either ADM (sertraline) 50-200 mg/d or 10 CBT sessions. At 12 months, CBT responders had greater glycemic control than ADM (sertraline) [95].

Patients on CBT not only improve depression but are also highly adherent to diabetic medication and self-monitoring of blood glucose, which ultimately lowers HbA1c levels [96]. Recently, in an RCT by Safren et al., 87 T2DM participants received 9-11 sessions of CBT and were followed up for 12 months. The study concluded that patient adherence with CBT had 24.3% higher medication adherence (95% CI: -38.2 to -10.3; p=0.001), 16.9% greater self-monitoring of blood glucose adherence (95% CI: -33.3 to -0.5; p=0.043), and 0.63 units lower HbA1c (95% CI: 0.06-1.2; p=0.03) [97]. In the healthcare system, despite having effective treatment options for comorbid diabetes and depression, 50% of patients are unwilling to take treatment, especially psychological treatment, due to the lack of accessibility or inconvenience [98]. As a result, healthcare providers rely more on pharmacological treatment due to the high cost of face-to-face CBT interventions [98]. Most recently, in the medical field, healthcare professionals are focusing on non-face-to-face models of alternative psychological interventions, including ICTs, known as eHealth, digital mental health intervention, and computer-based diabetes self-management interventions [98-100]. A systematic review of ICT-based psychological interventions appeared to reduce depressive symptomatology without improving glycemic control effectively [98]. However, computer-based diabetes self-management interventions over 3,578 patients from 16 RCTs by Pal et al. showed minimal benefits on glycemic control as HbA1c was 0.2% (95% CI: -0.4 to -0.1) without any effect on depression [100]. These new interventions are rapidly advancing; the scientific evidence is recent and very limited. More studies and economic analyses are needed to compare these interventions with the traditional face-to-face model and integrate these treatments into clinical practice [98-100].

Strength and limitation

This article explained the morbidity and mortality of comorbid diabetes and depression, their outstanding improvement in both treatment and remission with former ADMs and newer atypical antipsychotics, and the adverse outcomes of these drugs. However, diabetes is a multifactorial disease, and in our study, we have discussed only depression as a risk factor, and the other factors are ignored. This study could not identify conclusive evidence regarding the substantial gaps in the effectiveness of ADMs and CBT in treating depressive symptoms and glycemic control. Another drawback of this study is that it was not able to show any associations at the genetic level although they have shared physiological, behavioral, and environmental relationships. An integrative approach is highly recommended to understand the shared etiology at the genetic level and the effective treatment modalities for comorbid diabetes and depression.

## Conclusions

Diabetes is one of the significant public health burdens among all chronic health problems. From years of analysis, numerous studies included in this article showed that the prevalence rate of comorbid diabetes (T1DM and T2DM) and depression increased tremendously. This comorbidity is strongly associated with amplified risk of morbidity, mortality, decreased quality of life, and increased hospitalizations and health expenditure rates. Suffering from chronic diabetes increases psychological distress in the diabetic individual and is quite stressful for parents as continual care is challenging in the pediatric age group. Nevertheless, 50% of patients remain undiagnosed or untreated, and some overlapping somatic symptoms further hinder the early diagnosis of depression in a diabetic individual. The clinical implication of this review article is to highlight the importance of early diagnosis and management of comorbid diabetes and depression with a multidisciplinary approach to reduce the public health burden. We believe that this article can serve as a tool to overcome the challenges by providing a unique approach to the connection between the two entities by highlighting pathophysiological association, long-term consequences, regular screening, and management options. It is vital to implement the required treatment modalities; however, data from several studies demonstrated that the efficacy of ADMs or psychotherapy for relief or remission of depression is inconsistent, as some studies showed that CBT is better than ADMs. Others mentioned that ADMs are more effective. Additionally, patients are unwilling to attend regular face-to-face CBT interventions due to high costs and lack of accessibility or inconvenience. Nowadays, healthcare professionals are interested in non-face-to-face models of psychological interventions using different technologies, and this field is growing. More studies should be sufficiently powered to improve the prognosis of comorbidities to detect both treatment-related and treatment-independent effects of depression improvement in a diabetic individual.
